# Systemic sarcoidosis mimicking malignant metastatic disease

**DOI:** 10.3402/ecrj.v2.26761

**Published:** 2015-08-05

**Authors:** Irena Hammen, David Lee Sherson, Jesper Roemhild Davidsen

**Affiliations:** 1 Department of Pulmonal Medicine, Odense University Hospital, Odense, Denmark; 2 Department of Occupational Medicine, Odense University Hospital, Odense, Denmark

**Keywords:** sarcoidosis, malignant metastatic disease, lung cancer, granulomatous diseases, bilateral hilar lymphadenopathy, systemic disease

## Abstract

We present a case of systemic sarcoidosis involving the liver, pancreas, lungs, mediastinal and intraabdominal lymph nodes and bones. Multiple organ system manifestations mimicked malignant metastatic disease. The diagnosis was established with clinical, radiological, and pathological findings after neoplasm was ruled out by pathological tests. The patient showed rapid symptom remission with systemic steroid treatment.

Sarcoidosis is a systemic disease with unknown aetiology characterised by pathological findings of non-caseating epithelioid cell granulomas (1, 2). Scandinavians and African Americans have the highest geographic and ethnic incidence. In the United States, an estimated incidence rate of five cases per 100,000 person-years for men and 6.3 per 100,000 person-years for women has been reported (1). Danes seem to be more commonly affected with an incidence of 7.2 per 100,000 person-years with around 400 incident cases per year (3). The disease commonly affects young and middle-aged adults and can involve multiple organ systems. The most typical presentation is bilateral hilar lymphadenopathy, pulmonary infiltration, ocular and various skin lesions. The liver, spleen, other lymph nodes, salivary glands, heart, nervous system, muscles, bones, and other organs may seldom be involved (1, 2, 4). Cases with multiple organ involvement sarcoidosis can present a diagnostic challenge because they mimic many other diseases including metastatic cancer.

## Case report

A 45-year-old never-smoking male of Danish origin, without any medical history, was referred acutely to our emergency department with abdominal pain, vomiting, and jaundice. He denied alcohol use and had no predisposition to cancer, pulmonary, or gastrointestinal (GI) diseases. As a gardener, he had no relevant occupational exposures. During the last 4 weeks, he had suffered from a lack of appetite, dull pain in the right upper abdominal quadrant, and had an unintended weight loss of 10 kg. He denied any respiratory symptoms, change in bowel habits, melena, or fever.

At physical examination, he was chronically ill with a body mass index of 18, jaundice and abdominal pain on palpation of the upper abdominal quadrant. Pulmonary and cardiac auscultation was normal and no exanthema was present.

Laboratory tests showed leucopenia of 2.50×10^9^/L (3.50–8.80×10^9^/L) and increased liver enzymes tests with alanine transaminase of 441 IU/L (10–70 IU/L), alkaline phosphatase of 194 IU/L (35–105 IU/L), bilirubin of 196 µmol/l (5–25 µmol/L), and lactate dehydrogenase of 218 U/L (105–205 U/L). Pancreas amylase, gamma-glutamyl transferase, C-reactive protein, haemoglobin, thrombocyte concentration, calcium, and creatinine were within normal limits. Because of the liver involvement, hepatitis was suspected, but serology test for Hepatitis A, B, C, HIV, and Leptospirosis were negative. There were normal immunoglobulin subgroups and autoimmunological tests, including anti-nuclear and anti-neutrophil cytoplasmic antibodies, which were negative. However, an increased blood angiotensin-converting enzyme (ACE) above 88 U/L (8–53 U/L) was observed.

Lung function tests showed normal ventilation and diffusion parameters with forced expiratory volume in 1 second (FEV1) of 4.4l, corresponding to 102% of predicted; forced vital capacity (FVC) of 5.7l, corresponding to 105% of predicted; and lung diffusion of carbon monoxide (DLCO) corresponding to 85% of predicted. Total lung capacity was not measured.

**Fig. 1:**
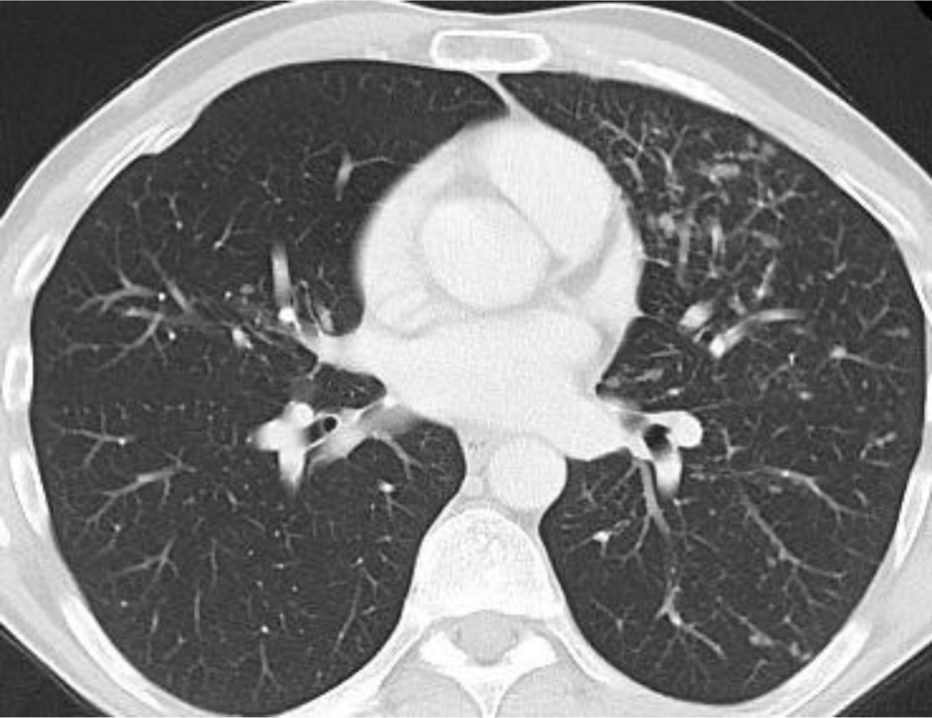
CT Thorax showing bilateral pulmonary nodular infiltrates.

**Fig. 2:**
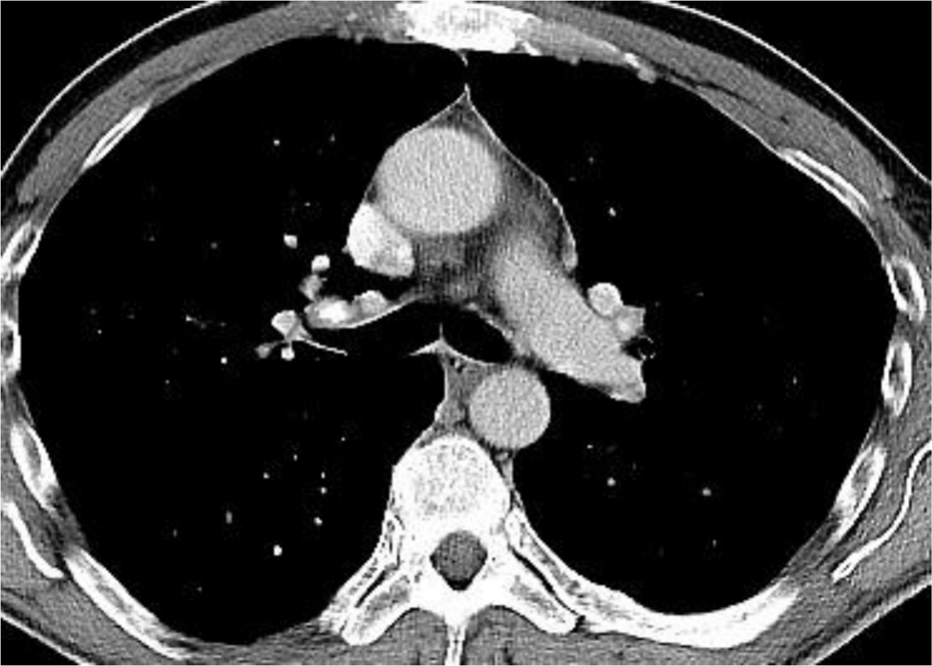
CT Thorax showing mediastinal lymphadenopathy.

**Fig. 3:**
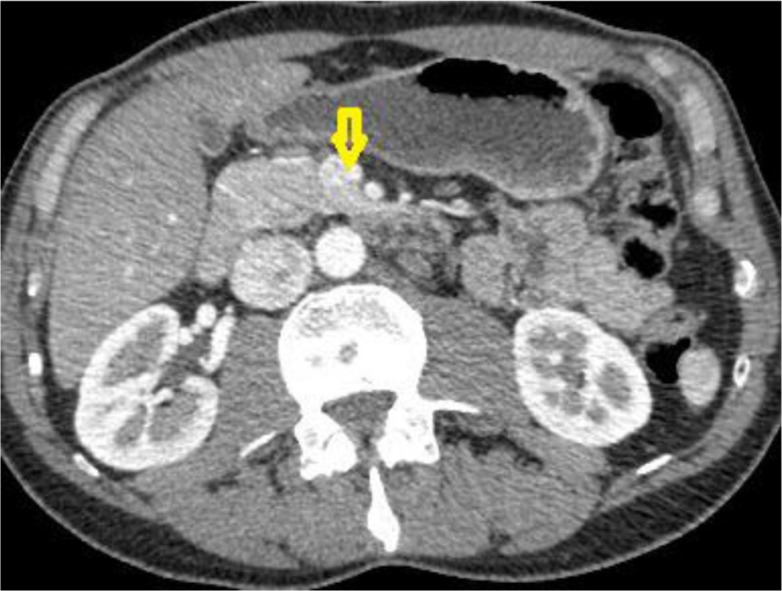
CT Abdomen showing lesions in the pancreas.

Because of the biochemical signs of hepatitis, an abdominal sonography was performed, uncovering lymphadenopathy in the lever hilus. The patient underwent computer tomography (CT) of thorax and abdomen, which revealed lesions in the pancreas, bilateral pulmonary nodular infiltrates, as well as mediastinal and intraabdominal lymphadenopathy (Figs. 1–3).

**Fig. 4:**
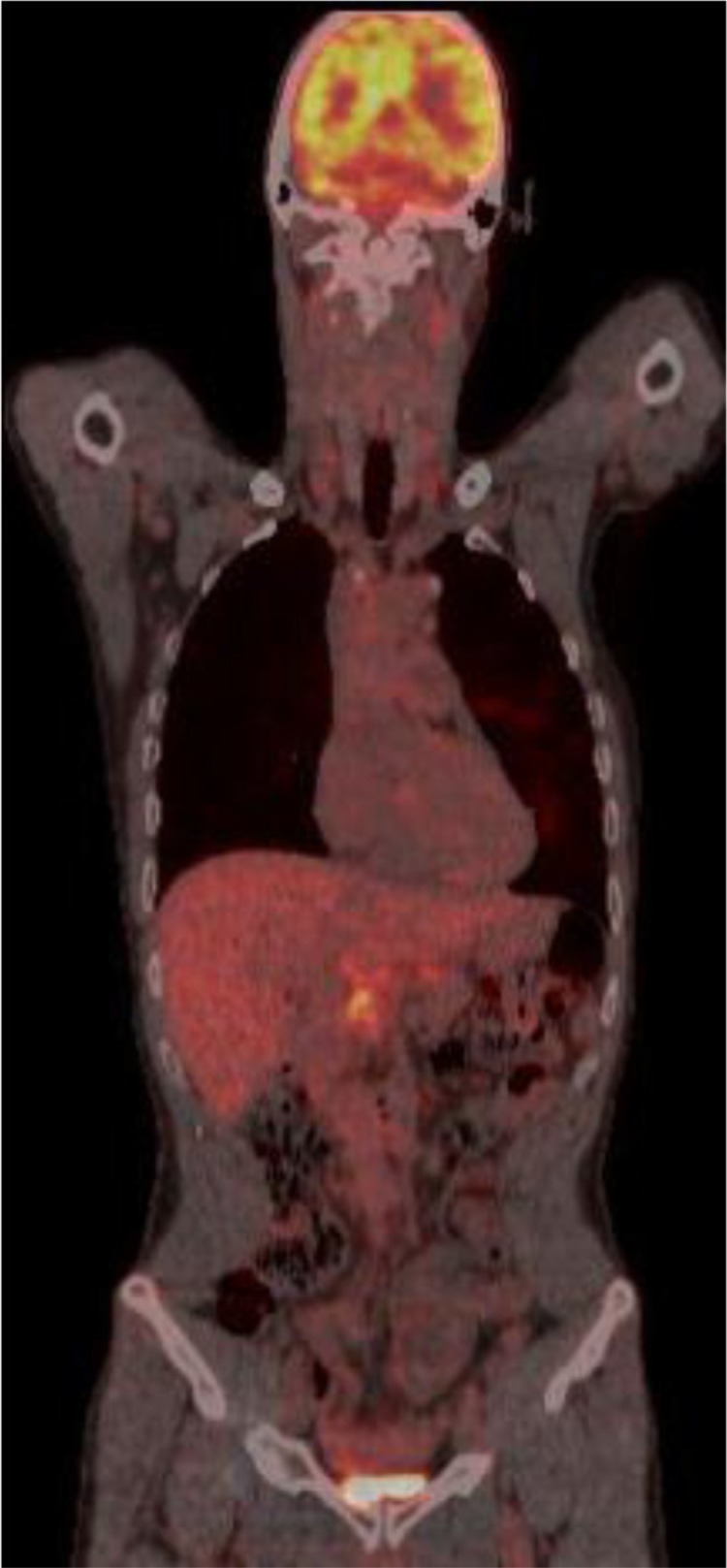
18-FDG PET scanning presenting multiple pathological lesions in lungs, pancreas, lymph nodes, and bones.

As metastatic cancer was suspected, a whole-body 18 flour-deoxyglucose positron emission tomography (18-FDG PET) scanning was performed, presenting multiple pathological lesions in lungs, pancreas, lymph nodes, and bones (Fig. 4).

A sonography-guided lever biopsy showed non-caseating granulomatous inflammation. The patient underwent further invasive procedures: bronchoscopy with transbronchial biopsy (TBB), bronchoalveolar lavage (BAL) with flowcytometry, endobronchial ultrasound (EBUS) with transbronchial needle aspiration (TBNAB) biopsy, and a bone marrow biopsy. None of the pathological tests revealed any malignant findings. Bronchoscopy was without any macroscopic abnormalities. BAL flowcytometry uncovered an overall lymphocytic inflammation with a proportion of 51%, an increased CD4/CD8 ratio of 5.4 without any eosinofilia or neutrofilia. Pathological evaluation of the samples from the TBBs demonstrated non-caseating granulomas and EBUS-TBNAB from the mediastinal lymph nodes showed unspecific fibrosis. Microbiological tests from the bronchoscopy specimens did not reveal any respiratory infection (no growth of bacteria in cultivation, negative tuberculosis tests including cultivation, negative PCR tests concerning *Legionella pneumophila*, *Mycoplasma pneumonia*, *Chlamydia psittaci et pneumoniae*, and *Pneumocystis jirovecii*).

We considered the following differential diagnoses: metastatic malignancy with unknown primary tumour, liver diseases (hepatitis, both infectious and autoimmune, biliary cirrhosis, and primary sclerosing cholangitis), infectious diseases (leptospirosis, AIDS, and tuberculosis), and autoimmune systemic diseases.

Based on the composite findings (increased blood ACE, various FDG metabolic active lesions in the PET-scanning, non-caseating inflammation in liver biopsy and TBBs, lymphocytic inflammation with increased CD4/CD8 ratio in BAL) and exclusion of other diseases capable of producing a similar pathological and clinical picture, a diagnosis of systemic sarcoidosis was well established.

The patient was treated with systemic prednisolone (0.5 mg/kg/day=37.5 mg/day), slowly tapering every third week (25 mg/day for 3 weeks, then 12.5 mg for 3 weeks), until a fixed dose of 7.5 mg prednisolone/day was reached. Three weeks into the treatment, blood tests showed normalised ACE and liver function parameters. At present we follow him in our respiratory outpatient clinic and plan to fully taper steroids according to the disease activity.

## Discussion

Sarcoidosis is a systemic disease, which in more than 90% of the cases involves the respiratory system and thereby manifests itself with pulmonary symptoms as dyspnoea and coughing (1, 2, 4). Non-specific constitutional symptoms such as fever, fatigue, malaise, and weight loss occur in about one-third of the patients as a result of increased immunological activity (4). In nearly one-third of the cases, the mediastinal lymph nodes are affected, giving rise to a sense of non-specific intrathoracic soreness. Multiorgan involvement is common while isolated extrapulmonary disease is rather rare and is reported in only up to 10% of the cases (1, 4). Cutaneous involvement is reported in approximately 25% (1, 4), neuro and cardiac manifestation in fewer than 10 and 5%, respectively, and ocular involvement in up to 83% of the patients with sarcoidosis (1).

The incidence of GI tract involvement is less than 1.0% and can mimic Crohn's disease or a GI fungal infection (4). Hepatic sarcoidosis is mostly observed in younger populations between 20 and 40 years of age, where some 30% may present clinical symptoms such as jaundice, nausea, vomiting, abdominal pain, and hepatosplenomegaly (5, 6). In some cases, untreated hepatic sarcoidosis can progress to liver cirrhosis with portal hypertension and cholestasis. This makes it challenging to distinguish hepatic sarcoidosis from hepatitis and cirrhosis, especially in cases of primary biliary cirrhosis and primary sclerosing cholangitis, where sarcoidosis can coexist (5). Hepatic involvement rarely causes hepatic failure, or increased mortality related to liver dysfunction (4).

Rare cases of pancreatic involvement have been reported although sparsely, for example, Shukla et al. reviewed 26 published cases with pancreatic manifestations from 1966 to 2007 (7). These cases included various abdominal symptoms as unspecific pain, weight loss, vomiting, and jaundice. Three patients even developed acute pancreatitis. Symptoms were due to organ infiltration and compression by enlarged lymph nodes (7).

Osseous involvement is relatively uncommon in sarcoidosis. The incidence is reported between 1 and 14%. Small bones of the hand and feet are most commonly involved, but other skeletal manifestations were also reported (8).

As this case report illustrates, due to multisystem involvement, systemic sarcoidosis can easily be mistaken for other systemic and especially metastatic disease (9, 10). It becomes even more challenging if the patient also has a history of previous or ongoing neoplasm. Clinical presentation with non-specific symptoms, such as weight loss, pain, dyspnoea, and cough, can be found both in case of malignancy and sarcoidosis.

The radiological presentation can also mimic malignancy. Typical findings in thoracic imaging (chest X-ray, CT-scanning, and high resolution CT-scanning) are hilar or mediastinal nodal enlargement (stage I), combined with parenchymal disease, that is, reticulonodular opacities, irregular nodular thickening in a perilymphatic distribution, nodules or large opacities, ground glass opacities (stage II) only parenchymal lung disease (stage III), and finally ongoing fibrosis with distorted lung architecture (stage IV). Due to a hypermetabolic state in active sarcoidosis, 18-FDG-PET scanning is not able to distinguish between sarcoidosis and neoplastic disease. Therefore, it is only recommended as a follow-up monitoring examination in patients with already confirmed sarcoidosis (11).

Granulomas are also sometimes found in lymph nodes draining malignancies and in the margins of malignant lesions including lymphoma and lung cancer. To make it even more complicated, patients with malignancy can at the same time develop sarcoidosis. The pathogenesis of cancer-associated sarcoidosis is not known, but has been observed with a coincidence of 14% in patients with malignancy (9, 10). The development of sarcoid-like disease, secondary to chemotherapy, is noteworthy, especially after therapy with the biological modifier alpha-interferon. However, such pathological reactions should not be mistaken with sarcoidosis (9, 12).

Further tests, such as an elevated ACE and CD4/CD8 ratio above 3.5 in BAL support the diagnosis of sarcoidosis. However, the gold standard is cytohistological confirmation. Biopsies can be performed from different involved organs, but the diagnostic yield varies significantly depending on disease manifestation. As almost 90% of sarcoidosis cases have pulmonary manifestations, TBB was previously preferred as the invasive examination of choice with a diagnostic yield ranging from 40 to >90% due to variation in inter-operator experience (1). As mediastinal lymphadenopathy appears in the acute and symptomatic stage, EBUS-TBNAB and oesophagus ultrasonographic (EUS) performed TBNABs are obvious examination modalities with an evident sensitivity of 89–94% (13) in recovering non-necrotizing granulomas and thereby exceeding TBB. In addition, EBUS- and EUS-TBNAB procedures are related to lower complication rates concerning pneumothorax and bleeding compared to TBB (13). Analysis from BAL fluid obtained during the bronchoscopy can be helpful, but may vary. An analysis of CD4/CD8 ratios and percentages of neutrophils and eosinophils revealed that a CD4/CD8 ratio of 4:1 or higher had a positive predictive value of 50% of sarcoidosis, and apparently only related to sarcoidosis (14). The same approach concerns a specific blood test as ACE, which is elevated in about 60% of sarcoidosis patients. These findings substantiate that elevated values of CD4/CD8 ratio and ACE are only indicators of sarcoidosis, but not diagnostic.

In conclusion, this case report illustrates that diagnosing systemic sarcoidosis can be a challenging puzzle due to the similarities between symptoms of systemic sarcoidosis and malignant metastatic disease. Thus, it is important to keep sarcoidosis in mind as a possible differential diagnosis of neoplasma, especially in non-smoking young adults with an abrupt disease course.

## Conclusion

In summary, the diagnosis of sarcoidosis requires compatible clinical symptoms, radiological findings, pathological demonstration of non-caseating granulomas, and exclusion of other diseases capable of producing a similar picture. Careful assessment particularly applies to cases where extrathoracic or multiple organ involvement is present.
